# Strategies to Build Trust and COVID-19 Vaccine Confidence and Engagement among Minority Groups in Scotland

**DOI:** 10.1007/s13753-022-00458-7

**Published:** 2022-12-12

**Authors:** Josephine Adekola, Denis Fischbacher-Smith, Thelma Okey-Adibe, Jamila Audu

**Affiliations:** 1 Adam Smith Business School, University of Glasgow, Glasgow, G12 8QQ UK; 2 Glasgow School of Business and Society, Glasgow Caledonian University, Glasgow, G4 0BA UK

**Keywords:** COVID-19, Vaccine engagement, Trust building, Minority group engagement, Science innovation, Scotland

## Abstract

As countries continue to deal with the global COVID-19 pandemic and its consequences, policymakers recognize that science, technology, and innovation (STI) practices offer a means of addressing many of the health problems that arise from the ongoing pandemic. Such recognition has given rise to many STI policy initiatives across various areas of science and policy, leading to a better understanding of coronavirus and the development of COVID-19 vaccines, treatments, and diagnostics. However, the practical implementation of vaccine and treatment strategies within local communities extends well beyond the laboratory. This study explored how misinformation and trust amplify or attenuate coronavirus and COVID-19 vaccine perceptions of those from ethnic minority groups deemed more susceptible to the impacts of the virus. Primary data in this study were collected in Scotland through semistructured interviews with 26 expert and nonexpert members from Scotland’s minority ethnic communities. The study findings show that risk perception is fluid and dependent on the information and evidential environment in which people find themselves. Misinformation, fake news, conspiracies, and trust or distrust (from prior experiences and historic practices) influence the perception of coronavirus and how risk messages are received, including the acceptance of coronavirus vaccines. This article reflects on Scotland’s approach to building trust and COVID-19 vaccine confidence and engagement based on the findings of this study, identifying areas of strength and areas for further improvement or research. The authors believe, as shown by our research, that vaccine engagement will be more impactful if developed by and with the public, and reflects public values, concerns, and priorities.

## Coronavirus Pandemic and Vaccine Engagement

The coronavirus pandemic has led to the loss of many lives and severely impacted economies worldwide (Nicola et al. 2020; Ozili and Arun 2020). Several policy measures have been put in place to curb the spread and impact of COVID-19, including national lockdowns, wearing of facemasks, washing of hands, and social distancing (Abideen et al. 2020; Ebrahim et al. 2020; Haider et al. 2020). These practices have been combined, in some countries, with an extensive vaccination program although the global pattern of vaccination has not been uniform (De Figueiredo et al. 2020). Following the declaration of the outbreak of coronavirus as a pandemic in March 2020, the Organisation for Economic Co-operation and Development (OECD) has continually tracked policy efforts made to mobilize scientific research to mitigate the impact of coronavirus. One such initiative is the Science, Technology, and Innovation Policy (STIP) COVID-19 Watch, an interactive database that provides open access to data on STIP initiatives. As of February 2022, over 900 policy initiatives had been reported by over 56 nations and the European Union under the supervision of the OECD Committee for Scientific and Technological Policy (CSTP). These STIPs are reported in several categories, including over a hundred policy instruments for the stimulus for Science, Technology, and Innovation (STI) systems; for diagnostics, therapies, vaccine research funding, and infrastructure support; and for governance arrangements to tackle COVID-19. There are also reported policy initiatives providing funding and infrastructure support for epidemiology research; science advice for policy; fundamental clinical research; and financial support for business innovation and the mitigation of long-term COVID-19 impacts.

One outcome of policy investments in diagnostics, therapies, vaccines research, and infrastructure support is a better understanding of the coronavirus itself, its mutations, and the development of COVID-19 vaccines, treatments, and diagnostics (Chen and Lu 2021; Kim et al. 2021). However, the COVID-19 pandemic has highlighted gaps in public management of vaccine uptake, especially around adult vaccination, leading to pockets of COVID-19 vaccine hesitancy across communities globally (De Figueiredo et al. 2020). Vaccine hesitancy can undermine vaccination coverage. It can lead to eight times the mortality rate over two years in countries with high hesitancy rates compared to countries with ideal levels of vaccination uptake, mainly if nonpharmaceutical interventions (for example, social distancing) are lifted (Mesa et al. 2022). Vaccine hesitancy is not new and has historically been linked to mistrust in experts, scientific institutions, and government, and has less to do with the misunderstanding of science (Stilgoe 2007), although hesitancy has been intensified by a rise in medical populism and conspiracy thinking as reported by Lasco and Curato (2019), Fischbacher-Smith (2021a), and Ullah et al. (2021).

Our aims in this study are twofold: (1) to explore the role of trust and misinformation in shaping COVID-19 and COVID-19 vaccine perceptions within Scotland’s ethnic minority communities; and (2) to reflect on Scotland’s approach to building trust and COVID-19 vaccine confidence and engagement, and to identify areas of strength and other areas needing further improvements or research. Understanding government strategies to build trust, engagement, and COVID-19 vaccine confidence will require a deeper understanding of the underlying issues of misinformation and trust within local communities. This is important in the context of Scotland, as data from Public Health England and Scotland suggest a considerable disparity in COVID exposure, vulnerabilities, and the least COVID-19 engaged groups (Public Health England 2020). This can be seen in the percentages of infection and deaths associated with ethnic minority communities (COVID Tracking Project 2020; Murugesu 2022). For example, Murugesu (2022) explains that 30% of all deaths between 24 January 2020 and 1 December 2021, in England of people aged over 30, are from ethnic minority groups, compared to 14% of those classed as White British. Thus, these statistical variations provide a highly relevant context in which to examine vaccine hesitancy within ethnic minority groups in Scotland.

## Misinformation, Trust, and Vaccine Hesitancy

Misinformation plays a vital role in shaping the inaccurate understanding of the hazards associated with vaccines. It has been described by Krause et al. (2020) in a pandemic era as “misinfodemic.” Krause et al. (2020) explained that misinformation shapes public perceptions of risk so that it can either intensify or attenuate the sense of concern about the hazard in terms of its probability of occurrence and consequences, which occurs at multiple levels. It is also challenging because risk is a term often used to mean different things, so it has become devoid of much of its meaning in widespread usage (Dowie 1999). Some of the underlying issues are seen to involve the magnitude of the misinformation, the extent of the trust shown in the sources of (mis)information, the mistrust in expert opinion, and the individual or group’s values and beliefs (see Fischbacher-Smith 2021a).

In the context of COVID-19, there has been significant uncertainty surrounding the nature of COVID-19 as an emergent disease. Thus, there is an extended scope for misinformation regarding the incubation period, the infectivity of the multiple emerging strains of coronavirus, the reinfection potential of the various strains, the mortality rate, and the effect of the virus on different population groups (Ioannidis 2020). There remains a lack of clarity around the effectiveness of the various disease control strategies as different nations have adopted different systems in the fight against coronavirus. Increased and frequent exposure to misinformation can negatively affect an individual’s adherence to official advice and willingness to get vaccinated against a virus (Roozenbeek et al. 2020). For this reason, Aven and Bouder (2020) called for caution regarding how decision makers refer to “evidence-led” coronavirus mitigation policies and guidance. Aven and Bouder (2020) argued that acknowledging uncertainties in characterizing and communicating risk makes it possible to distinguish between professional judgments about the nature of risk and those perceptions of the issues within social media and beyond. There is also a question about whether risk analysis, as a component of risk assessment, can adequately determine the probability and consequences of hazards where the failures are not random. Such assessments of both probability and consequence are often not based on a priori or statistical evidence but on expert (or even nonexpert) assessments of risk. These are seen to be weak in terms of their predictive validity (see Knight 1957). If we add to this the ambiguities surrounding the use of the term risk in practice, then it becomes a perfect storm in terms of how various publics understand risk-based messages.

Similarly, trust (or distrust) in information sources also has implications for the risk amplification process. For example, risk information from a trusted source may lead to risk amplification if it conveys information regarding increased danger or may lead to the attenuation of risk where there is a message of lacking danger. The opposite may occur where trust in the information source is lacking. In particular, public trust in government officials and other public health institutions has been essential in mediating public responses and any associated amplification or attenuation of risk information and emerging technologies (Frewer 2003). Also, trust in government and medical organizations is strongly associated with adherence to safety advice and vaccination uptake (Gilles et al. 2011; Prati et al. 2011; Quinn et al. 2013). Certain public groups, however, may perceive that a degree of vested self-interest might be associated with risk communicators responsible for protecting public health (for example, government officials), and this was evident in the ways that opposition to health officials in the United States was expressed by certain populist groups (Fischbacher-Smith 2021a). In the same vein, the government may have some responsibilities for risk generation, business or service continuity, and the management of risk regulators (Frewer 2003).

Misinformation and distrust can often increase vaccine concern and “delay acceptance or refusal of safe vaccines despite availability of vaccine services” (Macdonald 2015, p. 4163), termed vaccine hesitancy. Factors contributing to vaccine hesitancy are conceptualized with the 3C vaccine model as—confidence, complacency, and convenience (Macdonald 2015). Confidence concerns trust in vaccines’ effectiveness, the delivery system, and the policymakers’ motivations to decide on the needed vaccines (Macdonald 2015). Complacency is defined as low perceived risks of vaccine where preventable diseases and vaccination is not deemed a necessary preventive action. Lastly, convenience is the physical availability, affordability, willingness-to-pay, geographical accessibility, ability to understand (language and health literacy), cultural sensitivity, and appeal of immunization services that affect uptake. Other key factors leading to vaccine hesitancy are misinformation, sociodemographic context, and structural factors such as health inequalities and socioeconomic disadvantages (MacDonald 2015; Dror et al. 2020).

**Fig. 1:**
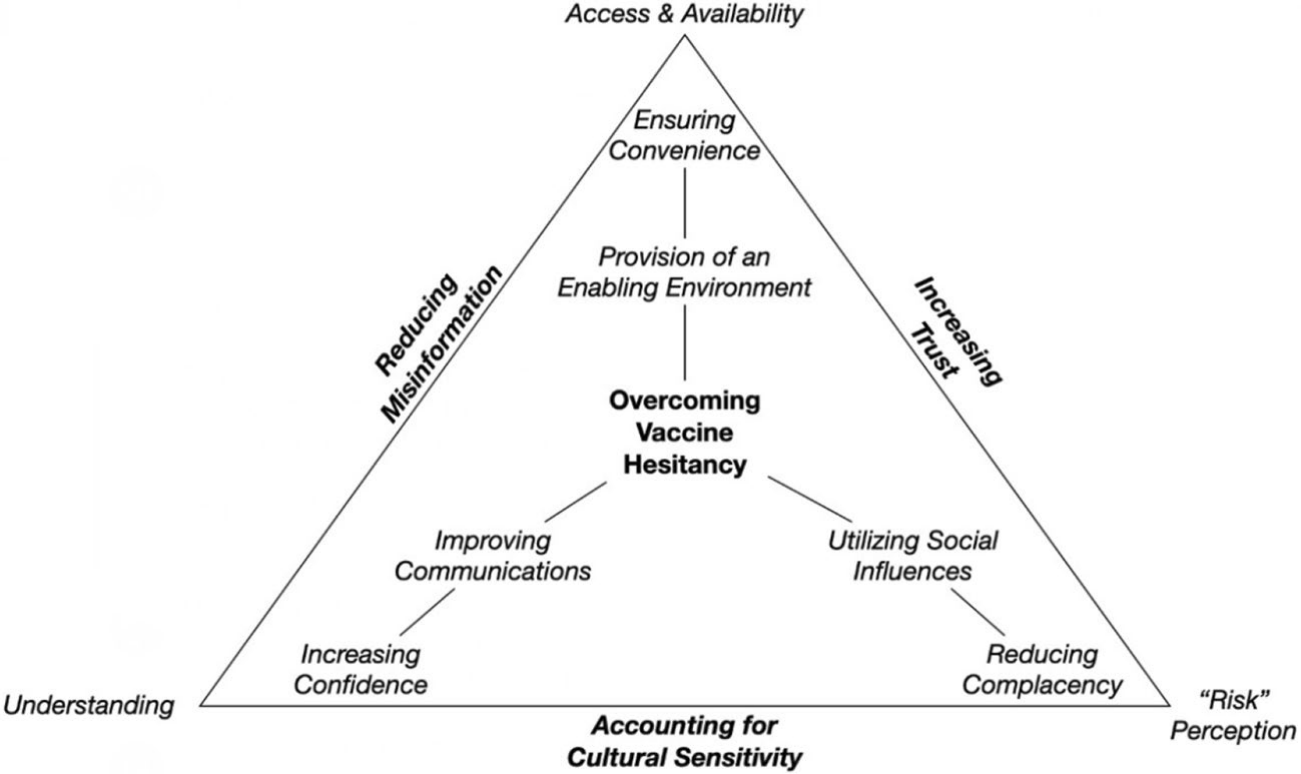
Elements of vaccine engagement. *Source* Fischbacher-Smith (2021b)

Figure 1 shows the various drivers that are seen to underpin the process of vaccine hesitancy that sit at the core of WHO advice. It provides the context within which misinformation and trust may shape the perception and understanding of risk. It also recognizes how access and availability may contribute to vaccine confidence and address issues around complacency and convenience. The core message here is that misinformation and trust affect how the public understands and perceives risk and the potential consequences. In combination with challenges around access and availability of vaccines, these factors could contribute to vaccine hesitancy, pose direct and indirect threats to human health, and potentially sabotage efforts to end the current COVID-19 pandemic or prevent future coronavirus pandemic risk.

Figure 1 also points to ways in which vaccine hesitancy may be overcome. It recognizes the need to create an enabling environment by ensuring that information is timely and relevant to its situational context (time and place). Improved communications can lead to an understanding of the costs and benefits associated with vaccine use, thereby increasing vaccine confidence. By using targeted information, especially through social media and influential bodies, risk perceptions may be changed (especially in terms of the hazards associated with vaccine avoidance relative to its use), and this may, in turn, reduce complacency. An essential outcome of these processes will be the reduction in misinformation (or, at the very least, the provision of an alternative narrative) and the potential to improve trust by recognizing and incorporating the values and ideals of different communities into the messaging.

Official information should be appropriately coordinated and void of conflicting messages. It does, however, need to be targeted communication that recognizes the different baseline perceptions that certain groups have and the presence of information accessible to those groups via online forums. Developing an ongoing relationship with different community groups could foster trust and encourage dialogue and debate on sensitive risk issues. Providing an evidentiary base that reflects the cultural norms of groups is essential for building confidence in people. For example, making raw data collected during the pandemic available for easy analysis will allow further insights into vaccination responses and reactions of members of ethnic minority groups. There is a need to form active partnerships with community groups while also recognizing and dealing with any historical and structural injustices in vaccine programs. According to Siegrist et al. (2003), trust influences public judgment of risk and how people accept associated costs and benefits. Therefore, it is a critical element in risk communication (Kasperson et al. 1992), particularly in how risk messages are decoded.

There is also a need to consider instituting or strengthening policies, laws, and programs that incentivize and create an enabling environment for vaccine uptake. Incentives could play a role in encouraging people to take the vaccines, which might take several forms of rewards—for example, giving the individuals some control or choice over where they take the vaccine and the brand of vaccine administered to them.

## Scotland’s COVID-19 Vaccination Strategy

The coronavirus vaccination program is the most extensive NHS immunization effort carried out in Scotland, following the advice from appropriate bodies such as the Joint Committee of Vaccination and Immunisation (JCVI). The JCVI advised that vaccine implementation should be flexible in terms of deployment, should mitigate health inequalities, involve carefully planned vaccine storage, transport, and administration, and ensure the availability of suitable approved vaccines for all within the Scottish NHS system. Therefore, inclusion and equality were embedded in Scotland’s national program, which involved working with stakeholders at all levels, including national bodies, local health boards, nongovernmental organizations, and community groups, to encourage vaccine engagement.

Scotland’s strategic framework sets out how Scotland would work collaboratively to “suppress the virus to the lowest possible level” (Scottish Government 2020, p. 16) while working to return to as normal a position as possible. Priority was placed on four key areas: (1) Suppressing the virus and protecting people, society, and the economy; (2) Protecting people involved in introducing new regulations, expanding testing capacities, regularly improving official advice, making further improvements to test and protect the health system, and providing support; (3) Defending society by placing a stronger emphasis on preparedness, investment in NHS mobilization and opening up of services, tackling inequalities, and keeping schools open also became a priority; and (4) Supporting the economy and working closely with businesses to provide tailored business support through funding, grants, and protective health measures.

Scotland’s COVID-19 vaccination program is focused on managing, distributing, and delivering COVID-19 vaccination to local communities as the UK government leads the purchase and supply of COVID-19 vaccines for the four UK countries. Advice is taken from the JCVI, and its implementation is adapted quickly. It is estimated that the program delivery will cost over 220 million pounds in 2021/2022 (Audit Scotland 2021). The first administered dose of the COVID-19 vaccine in Scotland was on 8 December 2020, after the first vaccine was approved by the Medicines and Healthcare Products Regulatory Agency (MHRA). Ten months later, the MHRA approved four COVID-19 vaccines (Pfizer-BioNTech, Oxford-AstraZeneca, Moderna, and Janssen). According to Audit Scotland (2021), COVID-19 vaccines have effectively reduced hospitalization and deaths from COVID-19 in Scotland. For example, the hospitalization rate in the unvaccinated group per 100,000 people over 60 years of age is 77 compared to 29 in the vaccinated group. With the age range of 16–59, the hospitalization number is 24 within the unvaccinated group compared to 8 in the fully vaccinated group.

Scotland’s vaccination program takes a very inclusive approach and has attempted to include many stakeholders, including community groups, academia, and the wider public. In the first phase of the vaccination program in Scotland, the country’s 14 Health Boards tailored their inclusive approach in line with six core themes—communications, flexible delivery models, accessible transport and clinics, engagement and coproduction, workforce, and data and evidence. This approach aligns with Fig. 1, and highlights the importance of enhancing understanding, developing and maintaining risk perception, and improving access and availability.

Within Scotland’s inclusive six core themes of vaccine management, the communication objective attempts to overcome the barriers to vaccine access and to increase vaccine confidence and engagement (Scottish Government 2022). To achieve this objective, the Scottish government set up a daily COVID-19 and vaccine update and a free national vaccination helpline to address concerns regarding COVID-19 vaccines and appointments. Essential communications were translated into over 30 languages on the Scottish National Health Service (NHS) information platform—NHS inform—and produced in accessible formats: easy read, British Sign Language, and audio. Some of this translation was carried out by people in the community who speak the language. All vaccination appointment letters sent out to the public have a QR code to enable the public to access further information, and there is a coproduced vaccine explainer video. There was also a series of questions and answers (Q&A) sessions at the national level for groups with low vaccination (Scottish Government 2022).

The inclusion strategy’s engagement and coproduction dimension aims to coproduce and tailor vaccine delivery for different community groups. To achieve a coproduced approach to vaccine inclusion, a National Vaccination Inclusive Steering Group was formed in 2020 and held regular meetings (every fortnight at the peak of the pandemic) to share learning between all stakeholders, including health boards and academia, nongovernmental organizations, and community organizations. At the pandemic’s peak, these meetings were held every fortnight and later moved to monthly meetings. There are also coproduced and tailored videos and health messages for minority groups such as unpaid care givers, refugees and asylum seekers, and faith groups. There was also an effort to work with local community groups to dispel myths about the vaccine.

The data and evidence dimension of the Scottish government’s inclusion strategy focuses on developing and sharing research, data, and evidence with health boards and partners. The establishment of and recommendations from an Expert Reference Group on COVID-19 led to a commitment to collect data based on ethnicity throughout the vaccination programs in order to embed this practice within the NHS culture. Data development by ethnicity provided insight into differences in vulnerability, exposure, and impact spread across the different ethnic groups in Scotland. Data collection by ethnicity also made it possible to identify engaged and less engaged groups. A regular update of vaccine uptake was extended to include uptake by the Scottish Index of Multiple Deprivation (SIMD) and ethnicity, as well as Public Health Scotland, which created a management information tool that enabled health boards to identify areas of low vaccine engagement.

Creation of flexible transport and models promotes increased vaccine accessibility for all willing to be vaccinated. This is achieved by identifying less vaccine-engaged groups from the health records and designing a vaccine engagement strategy that improves accessibility, such as drop-in, pop-up, and mixed model delivery. Vaccines are also administered in community venues such as places of worship, food banks, homeless shelters, and mass vaccination sites set up in city centers for easy access. Figure 1 recognizes the need to create an enabling environment through effective communications. It also highlights the need to creatively enhance the convenience of time, place, and uptake willingness. The goal of the accessible transport and clinic inclusion dimension is to overcome transport barriers to vaccination centers by paying attention to disabled and vulnerable groups. The Scottish government works with groups supporting disabled people to provide tailored services, which include developing a self-help guide on NHS inform detailing how to access drop-in clinics and setting up signposts to local clinics. Free travel to and from vaccination centers and interpretation support were provided at vaccination centers (Scottish Government 2022).

The workforce inclusion dimension provides a positive user-engagement experience for the public. This has led to mandatory equality and inclusion staff training of workers involved in the vaccination program (Scottish Government 2022). Military support (oftentimes from the UK government) was provided to help with planning and operational logistics, and Learning Disabilities Nurses were mobilized in some of the regional health boards, for example, in NHS Shetland (Scottish Government 2022). The Red Cross-National Volunteer Coordination Hub was established in Feb 2021 by the Scottish government and NHS Scotland to coordinate voluntary services and include dedicated inclusion and equality (Scottish Government 2022).

A key message here is that developing an inclusive vaccine program is critical to building trust in science and confidence in vaccines. This could also provide a degree of robustness to the critique of disinformation that arises out of specific social media influencers, although this is a complex relationship that speaks to the broader challenge of dealing with anti-science conspiracy theories.

## Methodology

Our research was partly funded by Glasgow Caledonian University (GCU); the University’s ethics committee approved the primary data collection methodology between March and April 2021. Data were collected using semistructured interviews, allowing in-depth discussion between the interviewer and interviewees. Participants included a collection of eight experts and 18 nonexpert members of an ethnic minority community in Scotland. The expert group interviewed in this study included five academics (at the level of professor and lecturer) and three public health experts. The study participants were recruited using snowballing and purposive sampling (Streeton et al. 2004; Tongco 2007; Creswell and Poth 2016). Fourteen participants (four expert and 10 nonexpert participants) were initially identified by taking advantage of existing social networks and relationships. Subsequent participants were identified using a snowballing technique. The number of participants (26) in this study is rationalized as saturation is believed to occur after 12 interviews (Guest et al. 2006).

Interviews were conducted via Teams or Zoom to comply with social distancing regulations in Scotland. Participants were all 18 years or over, lived in Glasgow and the surrounding areas, and self-identify as African, Asian, or other ethnic minority communities in Scotland. Twelve of the 26 participants who participated in the interview were females, and 14 were males. All interviews were fully transcribed, analyzed, and coded using thematic analysis (Braun and Clarke 2006) and followed a deductive approach (Reyes 2004). Table 1 outlines our study participants.

**Table 1:** Key demographics of participants

Nonexpert participants		Expert participants	
Category	Percentage (%)	Category	Percentage (%)
Age			
18–30	44.4	31–40	37.5
31–40	38.9	41–50	37.5
41–50	11.1	51–60	12.5
51–60	5.6	61–70	12.5
Gender at birth			
Female	44.4	Female	50
Male	56.6	Male	50
Marital status			
Married	55.6	Married	87.5
Single	27.8	Divorced	12.5
Single parent	5.6		
Civil partnership	5.6		
Widowed	5.4		
Ethnic background			
Black African	88.9	Black African	62.5
Asian	11.1	Asian	25
		Others	12.5

The participants were asked questions about their COVID-19 and COVID-19 vaccine perception, misinformation, trust, and vaccine engagement. The critical research question themes focused on: understanding of government strategy; nature of influential information sources; and the role of misinformation, fake news, trust, and distrust within vaccine hesitancy. The analysis was carried out and agreed upon by two of the researchers (JA and TO-A) using thematic analysis to identify key themes. These were initially coded individually, and the final theme was agreed upon between the authors (JA and TO-A).

Preliminary findings were discussed at a virtual event organized by the Women in Action group in Glasgow and funded by BEMIS (Empowering Scotland’s Ethnic and Cultural Minority Communities) on 26 June 2021, with 17 individuals from African and Asian communities in Glasgow. Key themes that emerged from the virtual event were the need to engage with members of the ethnic minority communities, working with grassroots community groups who have deeper reach into these communities, and communicating science and evidence to overcome conspiracies and misinformation. The feedback and questions generated during this virtual event were further incorporated into the research to strengthen the study analysis.

## Key Findings

The data presented in this article represent emerging themes around the perception of risk, misinformation, trust, and reasons for vaccine hesitancy within minority ethnic communities in Scotland.

### Risk Perception of Coronavirus

Analysis of interview transcripts revealed that, while all participants reported being very worried at the onset of the pandemic, 16 of the 26 participants were less worried than they were at the start of the pandemic. These 16 participants linked the reduced level of coronavirus concerns to factors such as familiarity with the virus as a function of knowing someone who was infected, knowledge of precautionary steps, vaccine uptake, and reduced reported deaths in the larger community. The following comments are typical of the responses made when asked about their concerns about the COVID-19 virus:

I am not as worried as I was last year, as I have friends who tested positive and have recovered well. (Nonexpert participant (NEP) 1)

At the present date, I’m less worried than I was last year because there is more information and more prevention methods that are more common to use now. (NEP 8)

At the present, I’m less worried because… the death rate of COVID; It’s actually decreasing now, it is not rising as before. I’m a bit less worried, hopeful. (NEP 2)

However, 10 participants who expressed an ongoing high level of coronavirus concern linked these worries to many factors. These factors include negative experience with the virus (including technical knowledge of the nature of the virus), existing personal health vulnerabilities, continued potential for exposure, the potential for emerging variants of the coronavirus, and especially where it had the potential to make the vaccines inefficient. Of particular concern was the potential for reinfection due to, for example, the emergence of a new variant, highlighted by six of the 26 participants. The following comments illustrate the nature of these concerns:

I’m worried. I’m anxious… because I was really sick when I had it, so I’m really worried—I’m really worried. (NEP 7)

There are reports that the available vaccines are not effect[ive] on some of the new strains of the virus. (NEP 18)

I already have the two vaccinations, which of course gives you some peace of mind or a certain level of peace of mind, but then again, the situation is quite uncertain with the emergence of the Indian variant with the usual measures whether we have the vaccines or not and that is a level of concern. (Expert participant (EP) 8)

One of the participants linked her knowledge of the risk of coronavirus infection to her level of concern:

Personally, I am worried, and in the sense that I know being a scientist who worked with coronavirus, I know the biology. I know the path the Coronavirus infection takes; when you have knowledge of something, you understand the risk, the weight of it, and so from that perspective, I am worried. (EP 2)

This reflects a long-standing recognition that those with first-hand knowledge of a hazard may show a heightened level of concern about the nature of the risks that the threat might generate. Our analysis references religious belief, as five of the respondents ultimately believe in divine protection. One respondent noted that while she was worried, she felt that she was “covered in the blood of Jesus.” (NEP 4). However, two participants noted that proximity to the virus was influential in changing that perspective to the reality of COVID-19:

At the start of the pandemic, I don’t take it very seriously. But over time when people around me got infected, I begin to realize the severity of the pandemic. (NEP 15)

[Initially], we were not really seeing anybody close to us contract Corona and then within the second round like people close to you, you know you’re my dad, my auntie and my cousins had Corona so like it’s real. (NEP 4)

Here, visibility or proximity to the risk can be seen to shape the risk perception of coronavirus and its seriousness (Slovic 1992).

### Public Understanding of Risk and Mitigations

Nineteen of the 26 participants expressed the belief that they had some level of knowledge of the government strategy. Although, this is not a claim of a scientific or technical basis for that policy:

I have a good understanding of the government and health authority strategies on the virus.… These information sources have informed my understanding of government strategies and about the virus generally. (EP 2)

I understand the government strategy in terms of restrictions and how they are currently being lifted. (NEP 1)

Four participants also noted inconsistencies in the government risk messaging and policy priorities that led to a limited grasp of the government strategy. This is reflected in two of the quotes below:

I don’t understand how they [the UK government] shut the borders so late… Put [on] the mask, sanitize all of that, but yet you kept the borders open. That to me just does not make sense. (NEP 5)

I think there is a good level of information coming from the government. However, sometimes that information is rather confusing and overlapping and I think that’s where the confusion occurs, and I sometimes think it’s perhaps a case of too much information that confuses individuals. (EP 3)

This suggests that there might have been confusion between the Scottish government’s response to coronavirus (where health is a devolved issue) and the UK government’s policies. Perhaps not surprisingly, these issues arise from the different priorities of the devolved administration relative to the UK, and the relative perceived “ownership” of specific policy issues. For locals in Scotland, there are two forms of government messaging—one from the Scottish government, which has responsibility for health care management, and another from the UK government (Cushion et al. 2020).

The participants were asked how they thought coronavirus fake news and misinformation influenced perception of coronavirus and its seriousness. All the participants suggest that fake news and conspiracy theories influenced the perception of coronavirus and COVID-19 vaccines within minority ethnic communities. For instance, one of the participants explained that:

I’m a Christian, and I heard 666, and I was like definitely not. I’m not wanting to take this vaccine… Taking the 666 means that like you’re rejecting God, or you are going to join that family. So, I was a bit panicky.” (NEP 2)

When this thing breaks up first, we were told that this content of animal which is not lawful as a Muslim, you cannot take that jab. But when we find out that, that was not the case, so the imams start to do this sermon and tell people look we better take this thing because there’s nothing forbidden that we take. (NEP 6)

Religiously inspired conspiracy theories were not the only ones that prevailed within the respondent group, and there were comments made about the origins of the virus:

It is quite interesting when you look at the information about the history of the virus. It reportedly started from Wuhan in China, and before it could spread to other Chinese towns such as Beijing, it had already spread to the whole of Europe and America. This prompted some curiosity on whether the virus was created in the lab, as widely mentioned. (NEP 14)

However, the data show that these suspicions were heightened where there is an alignment of conspiracy theories with existing religious and social narratives, as expressed by the two participants below:

So, there are plenty conspiracy theories… To a greater extent affect people’s participation, especially in the taking of the vaccine. And things like that and those who trying to bring 666 and all that… People heard all that then…some of the men of God, many men of God, especially from Africa, to be questioning Bill Gate’s role in this virus. (NEP 5)

I also listen to a popular religious preacher who believed that the pandemic is a conspiracy theory and that the end time prophecy is coming to pass. Therefore, agreeing to take up vaccine or any measure related to preventing the virus will lead to something else. (NEP 14)

However, three participants explained that these conspiracy theories did not influence their risk perception of coronavirus:

I don’t believe in any of those myths and conspiracies. This information is baseless and are [sic] largely untrue since there is no evidence to back the claims. So, I don’t give attention to them nor believe in them. (NEP 17)

I’ve been listening to doctors and well experienced people in terms of the health care, and I think that has influenced my perceptions now of these successes. I don’t believe it’s the 666 anymore. (NEP 3)

Obviously, there’s conspiracy of end of life, 666 and so many other ones like it’s a way of reducing people and the likes of its a humanly manufactured disease… I don’t think that has influenced my decision in any way… Yeah, I’d say zero or maybe one—I will say—so that it does not be a total zero. (NEP 9)

Notably, the influence of conspiracy theories is not limited to nonscientists alone. For example, one of the African community scientists noted he had watched a previously recorded documentary about how a Pfizer vaccine led to the death of 11 children in the north of Nigeria in the 1990s (Wise 2001; Jegede 2007). Such negative prior perceived experience with one coronavirus vaccine producer plays a role in how they perceive vaccines from manufacturers in the form of anchoring bias.

### Trust, Distrust, and Coronavirus Vaccination

The participants were asked the extent to which they feel trust or distrust regarding stakeholders (at the international, national, and community levels) is essential in accepting or rejecting public health messages around the coronavirus pandemic. All participants noted that the perception of trust and distrust regarding different stakeholders, especially government and pharmaceutical companies, can play a fundamental role in how the public receives official safety advice, including the acceptance of coronavirus vaccines. Some of the typical responses for lack of trust related to prior experiences and historic practices from the participants are reflected in the quotes below:

The government has done a lot, but the inequality that has existed previously in healthcare delivery in the country breeds distrust in the black community hence the hesitance to embracing the COVID-19 strategies. (EP 6)

Let me be honest; I don’t know if I can trust the government enough—I don’t 100% believe that there’s no agenda behind vaccination. (NEP 3)

Although the routine coronavirus briefings from the UK and Scottish governments were seen as a commitment to transparency and an effort at trust-building on the part of the government with the public, the first quote above suggests past experiences with the health system have implications for that trust.

Trust/mistrust in vaccines was linked to many factors, including the countries in which the vaccine was produced. For example, one of our participants noted that there was more trust for vaccines produced locally, that is here in the UK. The respondent:

It’s not the vaccines; it’s the people making the vaccines. Some people say, “They are playing politics because this vaccine was not invented in the UK. It was invented in America, so I rather like the UK vaccine more than the American vaccine.” I rather like the vaccine that was made by us in the UK than those vaccines that are made in America. So definitely, it’s a trust issue. (NEP 6)

Three of the participants attributed other trust-related factors to the lack of public trust in the way and the speed that the coronavirus vaccine was developed as well as with trust in the vaccine manufacturers themselves and their testing protocols:

Tell me one particular vaccine that has been produced in the past that was not first tested on animals before producing them. Do you know any? (NEP 5)

Closely related to this is lacking accountability in the event of adverse outcomes arising from the COVID vaccines, as reflected by two participants:

The fact that they also brought out laws on you can’t hold them accountable if something happens to you… if it’s completely okay and you know nothing is going to happen to us, why are you bringing out laws. (NEP 4)

[I saw] a documentary showing Pfizer asking for indemnity, if Pfizer is sure, why are they looking for immunity from the government? “in cases of unexpected outcomes…” if they are sure of what they are doing. I am worried and that’s why I don’t trust it (vaccine). (NEP 1)

Another respondent referred to the requirements for quarantine and the costs it generated, even after taking the vaccine, fuelled additional distrust in the effectiveness of some COVID-19 policies, as reflected in the below comment:

After the huge amount of money that has been spent on getting people vaccinated. If I am leaving this country today, I will still have to do the test, and it is also mandatory for me to quarantine when I return to the country. If you believe in the vaccine you are administering on the people; there is no point in requiring me to conduct the test again. (NEP 18)

Furthermore, another participant highlighted how conflicting information and opinions from scientists also contributed to a lack of trust in the scientific underpinnings of the advice:

There are two opposing views about the virus from scientific sources. For example, there was news going around about face masks can prevent the spread of the virus. Some groups (scientists) advocate that people should not stay at home, that the virus stays and spreads more indoors. Others (scientists) were of the view that people should stay at home, staying apart with two-meters distancing. (NEP 11)

The above statement reflects two things: (1) a hundred years elapsed between the flu pandemic and the COVID-19 pandemic, so practical experience was lacking; and (2) while much had been learned about the DNA of coronaviruses, the relative effectiveness of masks, hand washing, and social distancing was in its infancy as it applied to COVID-19. The same is true about knowledge of therapeutics, vaccines, and mechanisms of virus spread, not to mention the ecology of spread from animals to humans. These reflect the challenges in providing categorical evidence for problems with high uncertainty levels and the impact that such disagreements within the scientific community can have on public understanding of the issue. The quote from respondent NEP 11 also highlights how different forms of information can coalesce to generate distorted insights into the nature of the problem. Thus, the combination of elements, some factually correct, can combine with other information to create a different narrative around the nature and significance of scientific advice.

### Vaccine Engagement and Hesitancy

Regarding vaccine uptake, our data analysis provides mixed evidence, which suggests that there was hesitance in some quarters in the uptake of vaccines but that it was not a universal issue. At the time of the interviews between March and April 2021, 18 of the 26 participants had taken or intended to take the vaccine. According to one of the participants who had taken the vaccine:

Yes, I have taken the vaccine. It is a policy requirement, although voluntary, in my workplace. In addition to that, I believe prevention is better than cure, and everyone’s safety is important. And let me reiterate it that even if it were not part of my workplace conditions, I would still have taken the vaccine. (NEP 18)

However, the remaining eight participants were undecided, had not, or will not take the vaccine when offered. There were concerns expressed around tales of adverse reactions (fuelled to some extent by media reporting of a small number of blood clots arising from the vaccine), perceptions of differences in racial treatment, and the perceived lack of transparency from the government.

I’m not sure if I will take the vaccine or not… I heard that people took this vaccine, and some died… and also, there is this issue that it is going to affect your fertility basically, and I want to have a baby in the future… and it’s going to affect my fertility, which I would not want to happen. So that’s why I’m a bit sceptical about this whole vaccination. (NEP 2)

I think now; it is 50/50 because what happened is that my partner has got somebody that died from taking the vaccine. The person had a blood clot, so now we are worried, so for me, I’m worried, how would I react to this vaccine? I believe the COVID vaccine was so sudden, and I won’t want to take the vaccine for now. (NEP 7)

These concerns about the hazards associated with the coronavirus vaccine point to residual issues around the public understanding of risk in specific communities and should be acknowledged by government agencies charged with public health communication.

## Discussion and Implications for Future Public Health Communication Policies

This study’s findings highlight several issues that could usefully be incorporated into future public health strategies around risk communication for communicable disease outbreaks.

One of the key findings in this study is the argument that risk perception is fluid and varies depending on contextual factors. This highlights the need for a multilevel approach to communicating hazard-related issues and the rationale for the policies used to address those hazards. Analysis of interview transcripts revealed that while all participants reported being very worried at the onset of the pandemic, this concern lessened for some during the pandemic. This fluidity of risk perception depends on several factors, including the evolving state of information, knowledge, and understanding of the coronavirus and its treatment (Adekola 2019). Schneider et al. (2021) argued that risk perception is not static but one that changes as the situational context changes. For example, Schneider et al. (2021) noted a rise in risk perception when infection rates rise combined with the national lockdown and the emergence of a new coronavirus variant.

The Scottish government’s inclusion strategy aims to overcome the informational barriers to vaccine access and increase vaccine confidence and engagement. A key lesson for the future is to identify the critical communication nodes, especially those involving social media, and tailor communication to address the concerns within the groups influenced by those nodes. The Scottish government COVID-19 vaccine update and a free national vaccination helpline attempted to address the fluid nature of risk perception. This approach needs to be developed further to provide actionable intelligence to policymakers about the crucial communication channels for critical groups within local populations. The fortnightly meeting of the National Vaccination Inclusive Steering Group at the peak of the pandemic in 2020 helped stakeholders to share the learning that can be incorporated in vaccination implementation in real-time by relevant authorities. However, the extent to which such learning is communicated within the broader communities is beyond the scope of this study and remains an open question that deserves further scientific and policy attention.

The data suggest that fake news and conspiracy theories influenced the perception of coronavirus within minority ethnic communities. Suspicions were heightened where there was an alignment of conspiracy theories with existing religious and social narratives. Again, this points to the role that heuristics (particularly anchoring and representativeness) can play in shaping the perception of risk and uncertainty (Tversky and Kahneman 1974). The danger is that conspiratorial views can harm trust in government agencies, where evidence-based advice may be ignored or undermined, and this is especially problematic when those views are articulated by elected politicians.

However, the data and evidence dimension of the Scottish government inclusion strategy provided insight based on ethnicity into differences in vulnerability, exposure, and impact spread of the virus across the different ethnic groups in Scotland. This collection of data and evidence is a powerful tool that will help address some of the social narratives and conspiracies. It also helped identify areas of low vaccine engagement and created a better opportunity to research and understand the reasons for the hesitancy, which often centers on frequent exposure to misinformation (Roozenbeek et al. 2020). The insight generated by the data and evidence collection further presents an opportunity for more tailored research and opportunities for enhanced understanding that will better shape communication and engagement at the local level.

Our data reveal that the perception of trust and distrust (arising from prior experiences and historic practices) for crucial actors in a public health emergency, especially government and pharmaceutical companies, plays a fundamental role in how the public responds to official safety advice, including the acceptance of coronavirus vaccines. The routine coronavirus briefings from the government, a communication dimension of the inclusion approach, were seen by the respondents as a commitment to transparency and an effort at trust building on the part of the government with the public.

Our interview data also suggest that trust or mistrust in vaccines was often linked to the countries in which the vaccine was produced. For example, there was more trust for vaccines produced in Western countries when compared to those from non-Western countries. In this context, providing individuals with a choice in the type of vaccine they are given is a crucial step to addressing hesitancy linked to their trust in the source of the vaccine. There are, of course, challenges here regarding the availability of vaccines at critical points in a pandemic, and this raises a question about the capacity of “trusted” sources to respond quickly to an emergent pandemic.

Trust in core science was also seen as a crucial issue in vaccine hesitancy and often emanates from disagreements within the scientific community. The communication of the evidence base and the areas of potential disagreement are essential in building a coherent approach to addressing concerns about the burden of proof in emergent problems such as COVID-19. There remains, however, a fundamental problem associated with the scientific literacy of some sections of society, which has allowed medical populism to generate distrust in scientific evidence (Lasco and Curato 2019; Fischbacher-Smith 2021a; Fischbacher-Smith 2021b).

The interaction of multiple levels of misinformation and trust affects how the public accepts, delays, or rejects information about coronavirus risk; this shapes, in turn, the nature of vaccine hesitancy reported within some communities. These findings support existing studies showing that misinformation, fake news (Smith and Marshall 2010; Lockyer et al. 2021), and trust issues (Quinn et al. 2013; Vergara et al. 2021) contribute to vaccine hesitancy.

Regarding vaccine uptake, our data analysis provides mixed evidence suggesting some level of hesitance exists in some quarters in the uptake of vaccines. Therefore, engaging with communities provides opportunities to understand the nature of concerns, and these concerns can be used to frame the vaccine hesitancy dialogue with and within the community. These concerns could act as a means of responding to early warnings of future social and health crises.

## Conclusion

This study showed that conflicting and overlapping information from multiple scientific and non-scientific sources, the magnitude of information, sources/channels of misinformation, the role played by conspiracy theories, and inconsistencies between policy and risk messages all contributed to misinformation about the nature and severity of coronavirus. Trust or distrust in sources and channels of information, government, science, and pharmaceutical companies were key influencing factors on risk perception and the associated behavioral responses regarding adherence to safety measures, including the uptake of vaccine.

Scotland managed vaccine uptake within the general population well in most respects because its vaccination policy centered on six themes to overcome the barriers to vaccine uptake. This, to some extent, addresses the scope of the challenge to enhancing understanding using regular communication with the public through different means and languages, developing and maintaining risk perception through stakeholders’ engagement and sharing of knowledge, and improving access and availability using inclusive approaches and professionalism of the staff. However, the extent to which this approach was successful within the various communities in Scotland is beyond the scope of this study and remains an open question that deserves further scientific and policy attention.

Finally, evidence generated throughout the pandemic presents further opportunities for more tailored research and opportunities for an enhanced understanding of local needs to overcome barriers to vaccine uptake. There remains, however, a fundamental problem associated with the scientific literacy of some sections of society, which has allowed medical populism to generate distrust in scientific evidence.
